# Synthesis of Multilayered DLC Films with Wear Resistance and Antiseizure Properties

**DOI:** 10.3390/ma14092300

**Published:** 2021-04-29

**Authors:** Yucheng Li, Jun Enomoto, Yuki Hirata, Hiroki Akasaka, Naoto Ohtake

**Affiliations:** 1 Department of Mechanical Engineering, Tokyo Institute of Technology, 2-12-1, O-okayama, Meguro-ku, Tokyo 152-8550, Japan; enoenojunjuwar.0111@gmail.com (J.E.); akasaka.h.ac@m.titech.ac.jp (H.A.); 2 Institute of Innovative Research (IIR), Tokyo Institute of Technology, 4259 Nagatsuta, Midori-ku, Yokohama, Kanagawa 226-8503, Japan; hirata.y.ac@m.titech.ac.jp (Y.H.); ohtake.n.aa@m.titech.ac.jp (N.O.)

**Keywords:** DLC film, graphite, peelability, multilayered film, wear resistance, antiseizure, chemical vapor deposition, magnetron sputtering

## Abstract

Diamond-like carbon (DLC) films have attracted considerable interest for application as protective films in diverse industrial parts. This is attributed to their desirable characteristics, such as high hardness, low coefficient of friction, gas-barrier properties, and corrosion resistance. Antiseizure properties, in addition to wear resistance, are required during the die molding of polymer and polymer-matrix composite parts. Graphite films can be easily peeled because the vertically stacked graphene sheets are bonded via weak van der Waals forces. The present study demonstrates the fabrication of multilayered DLC/Cu films, where the Cu film functions as a catalyst for the formation of a graphite-like layer between the DLC and Cu films. The DLC/Cu film was synthesized on a Si (100) substrate via plasma-enhanced chemical vapor deposition and magnetron sputtering. The peelability, wear resistance, microstructure, texture, and cross-section of the film were experimentally analyzed. The results indicated a variation in the peelability with the deposition conditions of the Cu film that comprised particles with diameters of several nanometers. The DLC film at the interface in contact with the Cu film was transformed into a graphite-like state i.e., graphitized. The surface of the multilayered film exhibited antiseizure properties with the peeling of the upper DLC film. The multilayered film also exhibited wear resistance owing to the repeated appearances of a new DLC film. It is expected that the wear-resistant films with antiseizure properties demonstrated in the present study will be utilized in various industrial sectors.

## 1. Introduction

Hot-press molding and injection molding are extensively utilized mass-production techniques owing to the recent advances in material development and production technology. However, the demand for resin moldings is continuously increasing owing to desirable features such as a high production efficiency and low environmental load. These features are attributed to the demolding resistance that is generated by the release of the molded product from the mold. The demolding resistance originates primarily from the molded resin wrapped around the undercut portion of the mold. The occurrence of adhesion wear and seizure is accompanied by mold stain because the resin remains on the mold [1]. Generally, mold-release agents are utilized to decrease the release resistance [2,3] and to cool the mold; however, the application of these agents is associated with certain drawbacks. To satisfy the dual functions, a substantial quantity of mold-release agents is required per molding. Most of the utilized quantity is discarded, thereby resulting in a high environmental load. The application of these agents also results in the existence of core pins with low diameters and high mold-release resistances owing to the uncoolable and shrinkage force of the resin. Therefore, the use of mold-release agents cannot prevent the occurrence of seizures [4]. It is necessary to lower the environmental load by developing a surface modification technique that imparts antiseizure properties. An increase in the mold-release performance will allow the molding of complicated shapes. Furthermore, the elimination of secondary processing after molding and an alleviation of the mold-release defects will facilitate the optimization of the production efficiency.

Diamond-like carbon (DLC) films are extensively utilized in various fields owing to their high hardness, low friction, chemical stability, and high wear resistance [5,6,7,8,9,10,11]. The amorphous DLC films comprise sp^3^-bonded carbon of diamond with a tetrahedral 3D-network structure, sp^2^-bonded carbon of graphite with a hexagonal structure, and hydrogen [12,13]. The characteristics of DLC vary with the sp^2^/sp^3^ bonding ratio of C and H content [5,14,15,16]. DLC is typically prepared via chemical vapor deposition (CVD) [17] and physical vapor deposition (PVD) [18,19].

Graphite consists of graphene sheets assembled in a honeycomb structure via the covalent bonding of C atoms in sp^2^ hybrid orbitals [13,20]. It exhibits peelability and self-lubrication properties owing to the weak van der Waals forces, instead of strong covalent bonds, between the graphene sheets [21]. The transformation at the interface of DLC films in contact with Cu/Ni thin films into a graphene-like state has been reported in previous studies [22]. Cu is a transition metal with a closed-shell d-orbital, and the solid solubility of C in Cu is as low as 1–10 ppm. Furthermore, the interaction of Cu with C is extremely weak. The catalytic effect exerted by the dissociation of hydrocarbons and subsequent adsorption on Cu facilitates the formation of graphene [23]. The diffusion of the adsorbed C atoms to the surface is preferred in Cu over the precipitation reaction owing to temperature variations that occurs in Ni. The self-limiting effect of the reaction on the metal surface decreases the growth rate of graphene. This facilitates the synthesis of graphene with either a single layer or a controlled number of layers.

DLC, Cr, Cr3C2 and fluorinated materials films are utilized in demolding [24,25,26,27], and the coating of an injection-molding die with a DLC film induces an increase in the mold releasability [24]. Despite the application of those films, the molding of a highly viscous resin results in adhesion; consequently, the mold becomes dirty. The present study demonstrates the development of a functional surface film that not only retains the excellent characteristics of DLC but also exhibits wear resistance and antiseizure properties. A multilayered DLC/Cu film was synthesized via the alternate deposition of DLC and Cu films. Cu functioned as the catalyst for graphitization, and the outermost surface of the DLC film was partially graphitized at the interface with the Cu film. The synthesized multilayered film exhibited wear resistance and antiseizure properties owing to the alternate lamination of wear-resistant DLC films and peelable graphite layers.

## 2. Materials and Methods

### 2.1. Deposition

The DLC film was prepared via CVD. Here, a hydrocarbon gas (acetylene) was decomposed and ionized using a plasma at a low temperature (<500 °C) to deposit the film. The Cu film was prepared via a PVD technique: magnetron sputtering. Here, an inert gas (Ar) was introduced in a vacuum chamber, and used a Cu target as a cathode. Thus, an Ar glow discharge plasma was formed on the target surface. The generated Ar ions collided with the target, flicked the target atom, and deposited the atom on the surface of the DLC film.

**Figure 1:**
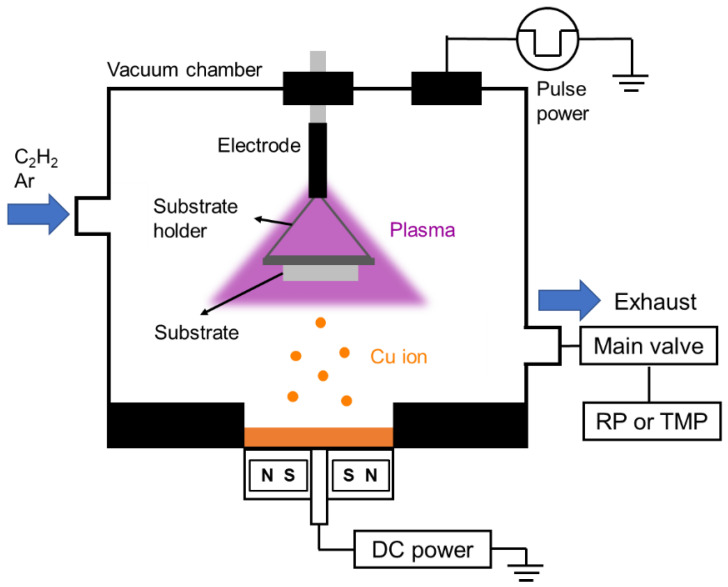
Schematic of the experimental setup.

The experimental setup for the deposition of the DLC and Cu films on a Si substrate is shown in Figure 1. The electrode plate was hung from the top of the vacuum chamber using a Y-shaped jig. The distance between the electrode plate and the substrate was 130 mm. The chamber pressure was initially decreased from atmospheric pressure to 10 Pa using a rotary pump (RP); subsequently, it was decreased to 5 × 10^−3^ Pa using a turbo molecular pump (TMP). The pressure inside the chamber was regulated, even after introduction of the gas, by adjusting the opening/closing degree of the main exhaust valve (MV).

Initially, Ar cleaning was performed for 30 min to increase the substrate temperature and eliminate the impurity films, such as the oxide film, on the substrate surface. Subsequently, the DLC film was synthesized using plasma CVD. The preparation time differed depending on the sample; therefore, the condition of each sample will be described later. The Cu film was synthesized via magnetron sputtering on the DLC film. A Cu target (99.9% purity, Kojundo Chemical Lab. Co., Ltd., Sakado, Japan) was placed at the bottom of the chamber, and a voltage was applied for 5 min. The shutter between the target and substrate was closed to eliminate the oxide film on the target surface via Ar pre-sputtering. The time at which the shutter was opened and voltage was applied was defined as the Cu sputtering start. Ar cleaning was not performed before the subsequent deposition of an additional DLC film on the Cu film. This was because the deposition was performed under identical vacuum conditions after Cu sputtering.

The Cu and DLC films were repeatedly synthesized using the above-mentioned method, based on the required number of films. The film-formation conditions are listed in Table 1, while the structures of the different samples are presented in Table 2.

The bottom, third, and fifth films were designated as DLC I, II, and III, respectively, while the second and fourth films were designated Cu I and II, respectively.

Initially, some of the samples in group A were subjected to heat treatment at 500 °C to confirm the effect of temperature on the graphitization of the DLC film in contact with the Cu film. DLC films may react with O_2_ and undergo degradation during heating in atmospheric conditions; therefore, the heat treatment was performed in a N_2_ environment. The gas was introduced at 2.0 × 10^3^ cm^3^/min for 30 min, thereby filling the muffle furnace with N_2_ before heating. The heat-treated and non-heat-treated samples were designated as α and β, respectively.

The 3-layer samples in group B were prepared under different Cu deposition times i.e., 25 (Cu (25)), 50 (Cu (50)), and 75 s (Cu (75)). The variation in the peelability with the thickness of the Cu film was investigated.

The Cu films were deposited under identical conditions for the 5-layer samples in group C, and the properties of each layer were investigated.

Finally, we designed an 11-layer sample with a wear-resistant multilayer DLC coating by increasing the Cu deposition time, based on the basic results of the experiment. This sample consisted of 5 DLC/Cu layers, thus it was able to be resistant to repeated seizure 5 times.

### 2.2. Film Evaluation

#### 2.2.1. Structural Properties

The particle shape and surface structure of the Cu film were observed using field-emission scanning electron microscopy (FE-SEM) (S-5500, Hitachi High Technologies). To confirm the structure of graphene or graphite on the DLC film at the interface in contact with the Cu film, the cross-sectional structure was observed using high-resolution field-emission transmission electron microscopy (FE-TEM) (JEM-2100F, JEOL Ltd., Tokyo, Japan).

The structural variations of the DLC film were characterized using Raman scattering spectroscopy (inVia Raman microscope, Renishaw, Tokyo, Japan) [14]. The excitation laser has a wavelength of 532 nm and a grating of 1800 l/mm. The laser output was 0.34 [mW], the irradiation time was 1 s, and the number of integrations was 20. For the analysis of the obtained Raman spectrum, peak separation was performed using analysis software (WiRE 4.0, Renishow, Tokyo, Japan). A superimposed Raman spectrum, with the G band at 1500–1600 cm^−1^ and D band at approximately 1350 cm^−1^, was obtained from the amorphous C film. The G band was derived from the vibrations, including the cyclic and chain-like vibrations, of the sp^2^ C-C bonds. The D band was derived from the defects formed during the expansion and contraction vibrations of the six-membered ring primarily at the edges of the graphite clusters.

An increase in the sp^3^-bond ratio induced a shift in the central wavenumber of the G band to the low-wavenumber side. Therefore, the integrated intensity ratio of the nuclear peak, i.e., I(D)/I(G), decreased. The full width at half maximum (FWHM) of the G band (FWHM (G)) represented the crystallinity of the graphite cluster. A highly crystalline structure corresponds to a sharp G band and a low FWHM(G). Furthermore, a disturbance in the structure induces an increase in FWHM (G) [28].

The Tuinstra–Koenig equation (Equation (1)) is related to I(D)/I(G) as an empirical equation for the grain size [15].

(1) $$\frac{I_{D}}{I_{G}}=\frac{560}{E_{L}{}^{4}}\frac{1}{L_{a}}$$

Here, $E_{L}$ is the energy of the excitation light [eV] and $L_{a}$ is the size of the crystallite [nm] in the six-membered ring. The equation denotes that I(D)/I(G) is inversely proportional to the size of the graphite cluster. Therefore, it was possible to evaluate the structure and crystallinity of C from the G-peak shift and either I(D)/I(G) or FWHM (G).

The microstructure of the DLC film at the polar surface was investigated using surface-enhanced Raman scattering (SERS) measurements. The SERS spectra were obtained based on the phenomenon of surface plasmon resonance (SPR). Metal (Ag) particles with a size of approximately 100 nm were adhered to the sample surfaces to calculate the Raman scattering intensity of the polar surface film. This allowed a detailed analysis of the film structure at the sample electrode surface [29]. The surface shape and roughness of the samples were observed using atomic force microscopy (AFM) (SPA300, Seiko Instruments Inc., Chiba, Japan) with a resolution in the nanometer range. The analysis was performed using the atomic force between the probe and samples.

#### 2.2.2. Mechanical Properties

An instant adhesive was attached to the samples to evaluate the peelability of each DLC layer. The properties of the film interfaces were analyzed after peeling.

To quantify the peelability, the peeling force was measured using a micro load cell (LTS-500GA, Kyowa Electronic Instruments Co., Ltd., Tokyo, Japan) attached to the pressurized part. The position was controlled using a servo press. A square sample (10 mm) was fixed to an acrylic plate using double-sided tape. A square cellophane tape (10 mm) was attached to the bottom of the jig, and the jig was contacted with the sample. The tensile load on the sample was measured, and the tape was peeled from the beginning until separation. The approach speed, pressing load, and release speed of the specimen and tape remained constant. The peeled area (A) was determined using image analysis software [30]. The energy required for peeling (U) was obtained from the peeling force and displacement, and the adhesion resistance was calculated using the following equation: U/A.

The tribological characteristics, such as the friction coefficient and wear resistance, were evaluated using a ball-on-disk (BoD) test, where the load was assumed to be applied during resin inflow. The vertical load owing to the weight was set to 0.5 N, and the rotation speed was set to 100 rpm. The horizontal load was measured seven times within one rotation using a bearing steel ball (Japan standard, SUJ2, TSUBAKI NAKASHIMA CO., LTD., Katsuragi, Japan) with a diameter of 6 mm. The median value of the seven measurements was considered as the friction coefficient for evaluation.

## 3. Results and Discussion

### 3.1. FE-TEM Observations of the Cross-Sectional Structure of the Deposited Film

**Figure 2:**
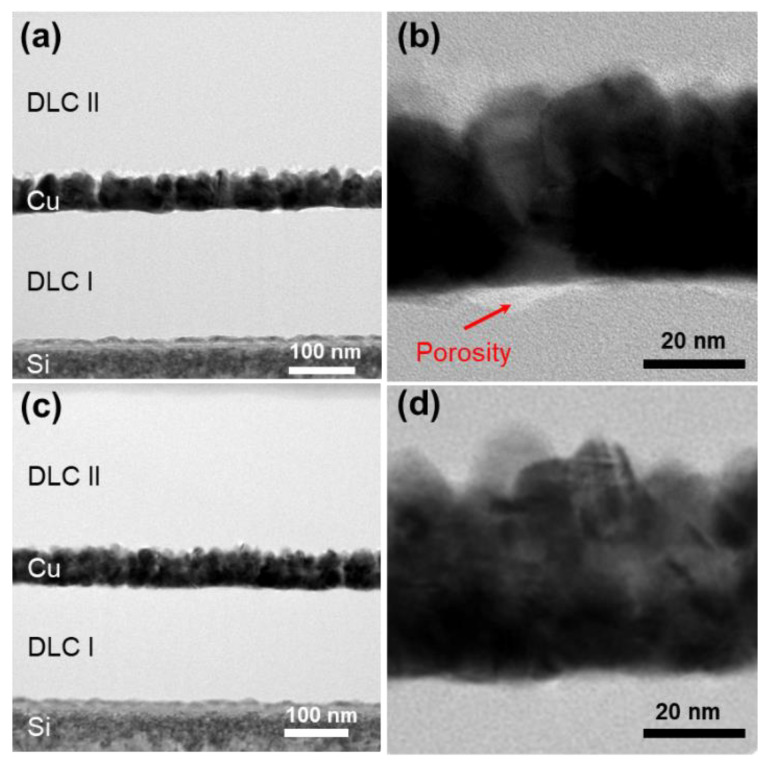
TEM (**a**) overview and (**b**) close-up view images of DLC/Cu (300)/DLC with heat treatment. TEM (**c**) overview and (**d**) close-up view images of DLC/Cu (300)/DLC without heat treatment.

The cross sections of the samples in group A, with and without heat treatment at 500 °C, were observed via FE-TEM, and the images are presented in Figure 2.

The thickness of the DLC II film formed on the Cu film was higher than that of the DLC I film formed on the Si substrate. This was attributed to the adhesion between the deposited surface material and the DLC film. Generally, DLC films with internal stress exhibit stronger adhesion to Si than that to Cu. This is because Cu is soft and susceptible to plastic deformation. Cu did not form carbides and bonds with C; therefore, the density of the DLC I film was higher than that of the DLC II film.

The existence of porosity between the Cu and DLC films was detected in the samples subjected to heat treatment; however, no such porosity was detected in the samples without heat treatment. This was attributed to the thermal expansion and contraction of the Cu film during the heat treatment and subsequent cooling, respectively. The extent of expansion and contraction for the DLC film was less than that for the Cu film. Boubiche et al. reported that a metal catalyst facilitated the clustering of sp^2^ bonds throughout the DLC film along the orientation of the metal nucleus with an increase in the temperature [31]. Ilie et al. reported that heat treatment induced a transformation in the orientation of the sp^2^ clusters in the a(amorphous)-C film from vertical to parallel to the substrate. Consequently, there was an increase in the size of the graphite clusters [32]. This was attributed to a decrease in the adhesion of the interface and the wear resistance under heat treatment.

**Figure 3:**
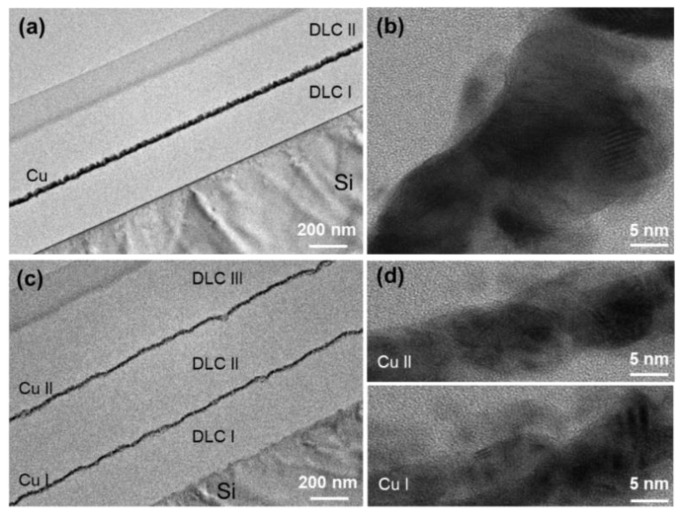
TEM (**a**) overview and (**b**) close-up view images of the 3-layer sample, DLC/Cu (50)/DLC, in group B. TEM (**c**) overview and (**d**) close-up view images of the 5-layer sample, DLC/Cu (25)/DLC/Cu (25)/DLC, in group C.

Figure 3 presents the TEM images of the samples in groups B and C i.e., DLC/Cu (50)/DLC and DLC/Cu (25)/DLC/Cu (25)/DLC, respectively. The sample interfaces presented different thicknesses, and the images in Figure 3a,c confirmed the 3-layer and 5-layer structures, respectively. The samples without heat treatment did not exhibit any porosity, unlike that for the samples subjected to heat treatment.

### 3.2. Structural Properties

#### 3.2.1. Surface Observation of the Cu Film Using FE-SEM

**Figure 4:**
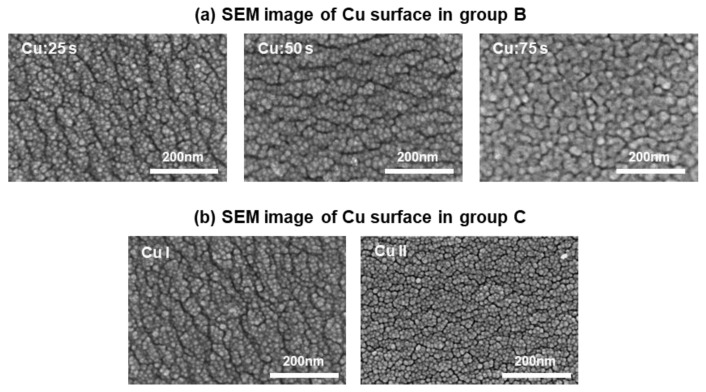
SEM images of the Cu surface for the samples in (**a**) group B and (**b**) group C.

**Figure 5:**
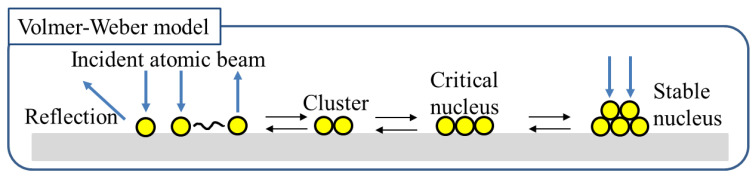
Formation of a nucleus in the Volmer–Weber model.

The surface structures of the Cu films, with different deposition times, for the samples in group B were observed via FE-SEM, and the images are presented in Figure 4a. The SEM images elucidated the effect of the thickness of the Cu film on the peelability. The particle size of Cu (25), Cu (50), and Cu (75) was approximately 15, 20, and 25 nm, respectively. The diameter of the Cu particles increased with an increase in the Cu deposition time. This was ascribed to the condensation of nuclei into a large nucleus based on the Volmer–Weber model of film formation. Figure 5 shows the formation process of the nucleus in this model [33]. The Volmer–Weber growth of polycrystalline films involves the nucleation of 3D islands on a substrate surface and the subsequent growth, impingement, and coalescence of islands to form continuous films [34,35,36]. The thin-film growth techniques and growth conditions affect the grain shape, grain size distribution, and distribution of the crystallographic orientation of grains [37]. The variation in the stress-thickness product for different film thicknesses is attributed to the adatom-surface interaction energy [38]. The high mismatch between the DLC surface and Cu in the early stage of deposition facilitated film formation according to the Volmer–Weber model.

We also analyzed the crystal structures of the Cu I and II films for the samples in group C using FE-SEM, and the images are presented in Figure 4b. The Cu I and II films were synthesized under identical conditions with a deposition time of 25 s. The particle size was approximately 10–15 nm, and no significant differences in the size were observed. However, the 3D structure and growth exhibited by the Cu II film was more prominent and more advanced, respectively, as compared to those exhibited by the Cu I film. The sputtered particles of Cu collided with the DLC film, and the kinetic energy of the sputtered particles increased the temperature of the collision surface and the energy of the outermost layer of the DLC film. Zhou et al. fabricated a 20 nm-thick Cu film on an 80 nm-thick DLC film using a vapor deposition technique. They conducted elemental analysis via Auger molecular spectroscopy in the direction of the film thickness. The results indicated the diffusion of C atoms into the Cu film [39]. Dwivedi et al. identified the presence of C nanoaggregates with a network-like structure on the surface of a C: H film formed on a Cu film. This indicated that Cu functioned as a catalyst and contributed to an increase in the sp^2^ bond content [40]. Therefore, the C atoms on the outermost surface of the DLC film diffused to form nanocrystal graphite.

#### 3.2.2. Microstructural Analysis of DLC Film Using Raman Scattering Spectroscopy and SERS

**Figure 6:**
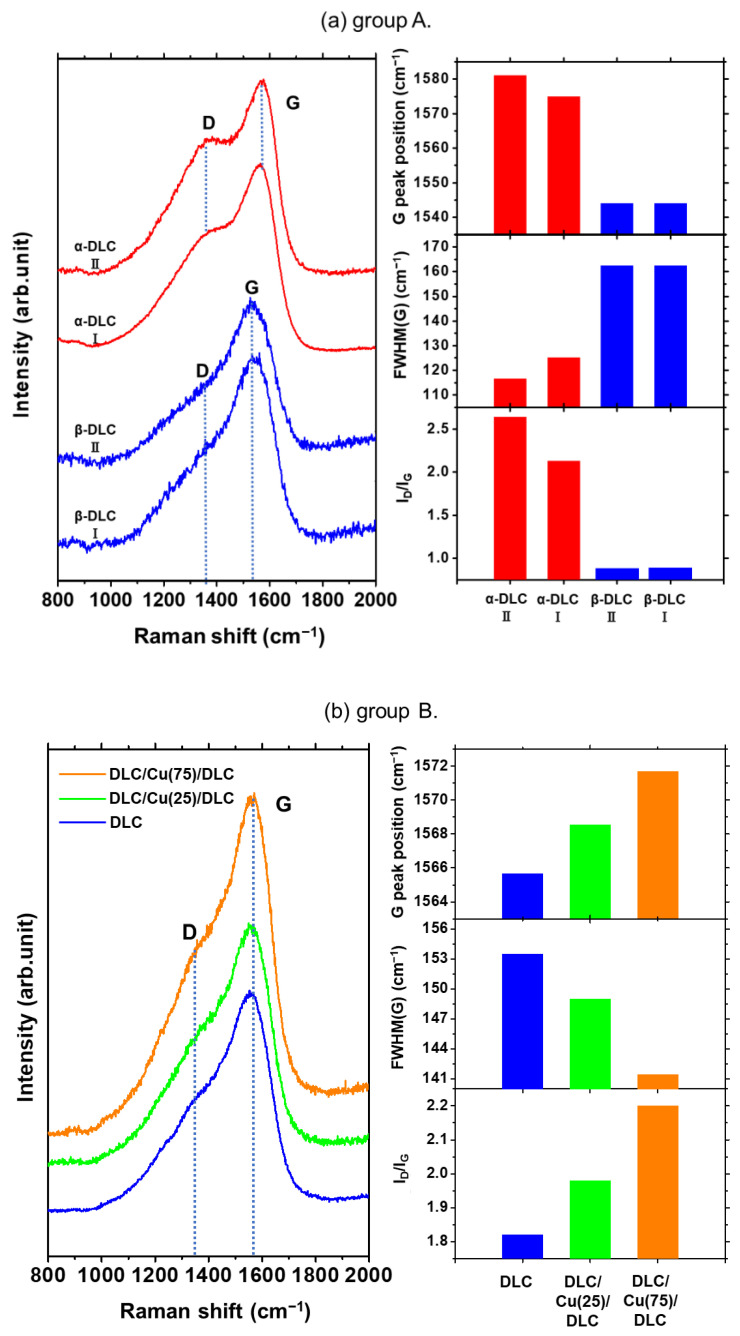

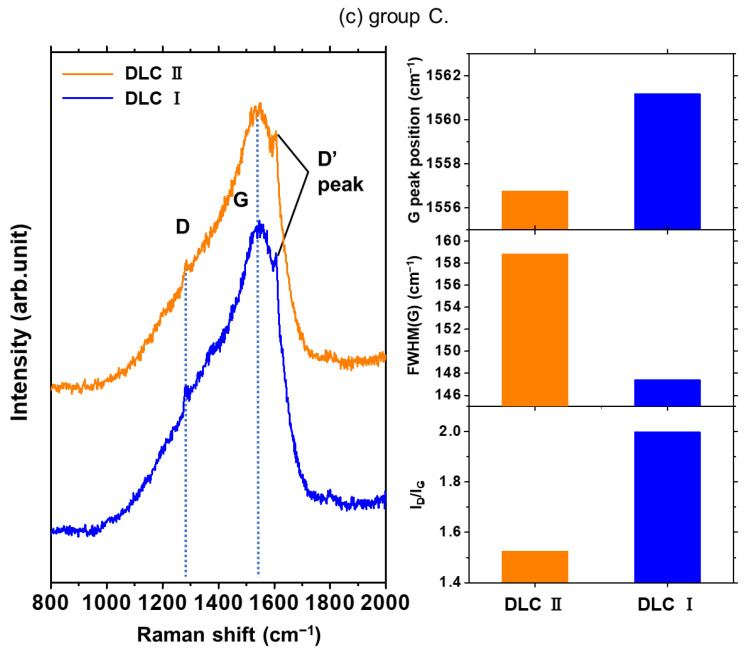
(**a**) Raman spectra and Raman parameters of the DLC I films for the samples in group A. (**b**) Raman spectra and Raman parameters for DLC/Cu(X)/DLC in group B. (**c**) Raman spectra and Raman parameters of the DLC I and II films, after peeling, for the samples in group C.

Figure 6a shows the Raman spectra of the samples in group A. Initially, the spectra of the heat-treated sample were analyzed. The DLC II film presented slightly split peaks, while the spectra of DLC I film was not distinctly separated. The central wavenumber of the G peak for the DLC II film exhibited a tendency to shift to the high-wavenumber side. Therefore, the FWHM (G) and I(D)/I(G) for the DLC II film were lower and higher, respectively, than those for the DLC I film. This indicated that the proportion of sp^2^ bonds was higher in the DLC II film as compared to that in the DLC I film. An increase in the crystallinity of the graphite clusters was detected, thereby suggesting a structure similar to that of nanocrystal graphite. The analysis of the spectra for the non-heat-treated sample revealed that the DLC I and II films presented one broad peak with superimposed D and G bands. In addition, the occurrence of graphitization was not detected. The spectra of the heat-treated and non-heat-treated samples in group A were compared. The central wavenumber of the G peak shifted to a higher level for α as compared to that for β. A decrease in the FWHM (G) induced an increase in the I(D)/I(G) proportion of sp^2^ bonds, and crystallinity of the graphite clusters. This indicated that heat treatment promoted the graphitization of the DLC film.

Figure 6b shows the SERS spectra of the samples for DLC/Cu (0), DLC/Cu (25)/DLC, and DLC/Cu (75)/DLC in group B. The samples under analysis were the DLC I films after peeling of the DLC II films. Although the deposition conditions of the DLC films were identical, the Raman spectra presented different features depending on the Cu deposition time. The central wavenumber of the G peak was ordered in the following sequence: DLC/Cu (75)/DLC > DLC/Cu (25)/DLC > DLC/Cu (0). The shift in the central wavenumber to the high-wavenumber side became more pronounced with an increase in the Cu deposition time. This was attributed to an increase in the proportion of sp^2^ bonds. The central wavenumber of the G peak for DLC/Cu (75)/DLC was 1572 cm^−1^. This indicated that the DLC film at the surface was transformed to a structure similar to that of graphite. The FWHM (G) was ordered in the following sequence: DLC/Cu (75)/DLC < DLC/Cu (25)/DLC < DLC/Cu (0). This result revealed that the crystallinity of the graphite clusters in the DLC film increased with an increase in the Cu deposition time. The integrated intensity ratio (I(D)/I(G)) was ordered in the following sequence: DLC/Cu (75)/DLC > DLC/Cu (25)/DLC > DLC/Cu (0). This result confirmed that the crystallinity of the graphite clusters increased with an increase in the Cu deposition time. It was inferred, based on the aforementioned results, that the DLC film in the surface film was graphitized with an increase in the deposition time of the sputtered Cu film.

Figure 6c shows the SERS spectra of the DLC I and II films, after peeling, for the samples in group C. Although the deposition conditions of the Cu films were identical, the spectra revealed differences in the peak intensities. Both the DLC I and II films presented a broad peak with superimposed D and G peaks in conjunction with other sharp peaks at approximately 1600 cm^−1^. This was attributed to the summation of the G and D’ peaks [16,41]. The D’ peak was ascribed to the vibration of the sp^2^ C-C bond of small graphite particles, thereby indicating the presence of nanocrystal graphite [16]. The structure of the DLC II film was more amorphous than that of the DLC I film owing to two factors. Firstly, the DLC film exhibited stronger adhesion to the Si substrate than that to the soft Cu film; therefore, the density of the DLC I film was higher than that of the DLC II film. Secondly, the adhesion of sputtered particles (Ag) exerted a significant influence on the Raman spectra, except for the nanocrystal graphite film of the outermost surface at a depth of approximately 5–10 nm. The graphitization of the DLC film on the outermost surface progressed significantly on the first-peeled surface. This was attributed to an increase in the substrate temperature with the progress of deposition, thereby resulting in a high-intensity D’ peak.

#### 3.2.3. Observation of the Surface Roughness Using AFM

**Figure 7:**
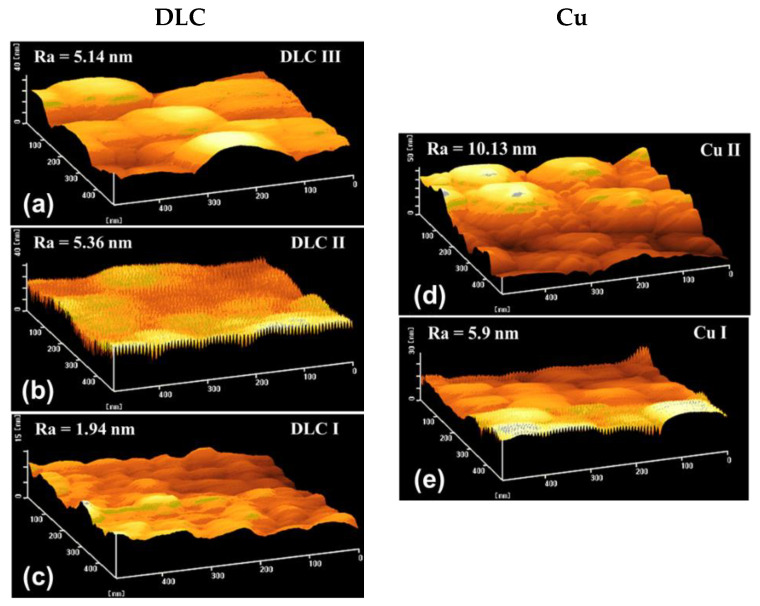
Surface shapes and roughness of the (**a**) DLC III, (**b**) Cu II, (**c**) DLC II, (**d**) Cu I, and (**e**) DLC I films for the samples in group C.

The surface shape and roughness of the samples in group C were evaluated using AFM, and the results are presented in Figure 7a–e and Table 3. Although the DLC films were deposited under identical conditions, the roughness and thickness of the DLC I film were significantly lower than those of the DLC II and III films. This was attributed to the high density of the DLC I film that adhered to the Si substrate. The surface roughness of the DLC II and III films were 5.36 nm and 5.14 nm, respectively. The Cu particles impacted the surface of the DLC II film during the formation of the Cu II film. This collision resulted in the higher roughness of the DLC II film as compared to that of the DLC III film. The roughness and contact area of the Cu II film were higher than those of the Cu I film. This was because the Cu II film was formed on the DLC II film that was rougher than the DLC I film. The extent of graphitization of DLC induced by the Cu II film was higher than that induced by the Cu I film. Therefore, the upper DLC/Cu interface was expected to exhibit high peelability.

### 3.3. Mechanical Properties

#### 3.3.1. Evaluation of Peelability via Measurement of Peeling Force

**Figure 8:**
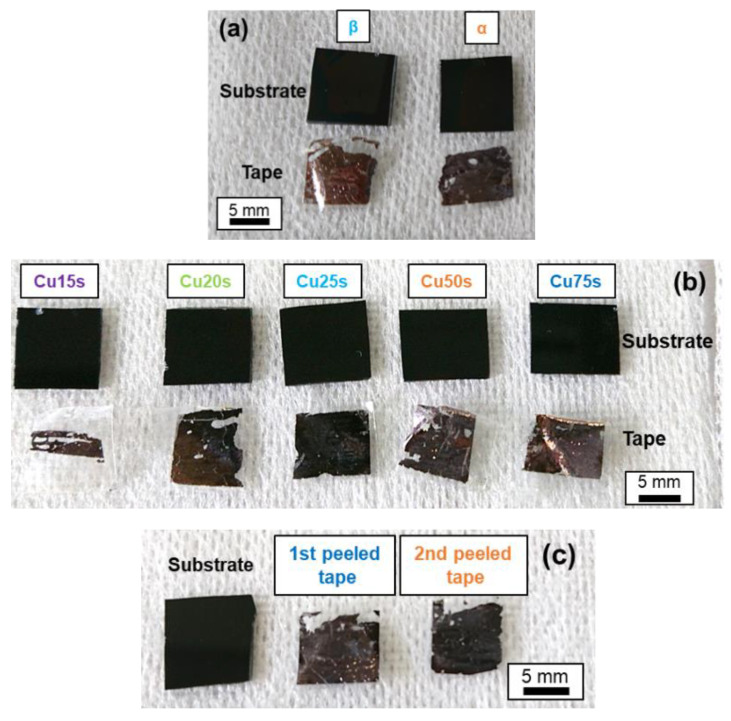
Appearance of samples in (**a**) group A, (**b**) group B, and (**c**) group C.

Cellophane tape was attached to the upper film of the prepared sample and peeled off to determine whether peeling was possible. As shown in Figure 8a,b, a brown Cu film was observed on the peeled surface and tape side, while the DLC film was observed on the substrate side of the samples in groups A and B. This indicated the occurrence of peeling at the DLC I/Cu interface. The positions of all the samples and tapes were fixed, and there were no significant differences in the measurement area. DLC/Cu (15), (20)/DLC exhibited incomplete peeling, whereas DLC/Cu (25)/DLC exhibited uniform peeling. Therefore, the optimal deposition time was determined to be 20–25 s. The brown Cu films adhered to the peeling tape side, while the glossy DLC films adhered to the substrate side of the samples in group C during the first and second peeling shown in Figure 8c. This confirmed the successful fabrication of a twice-peelable structure.

**Figure 9:**
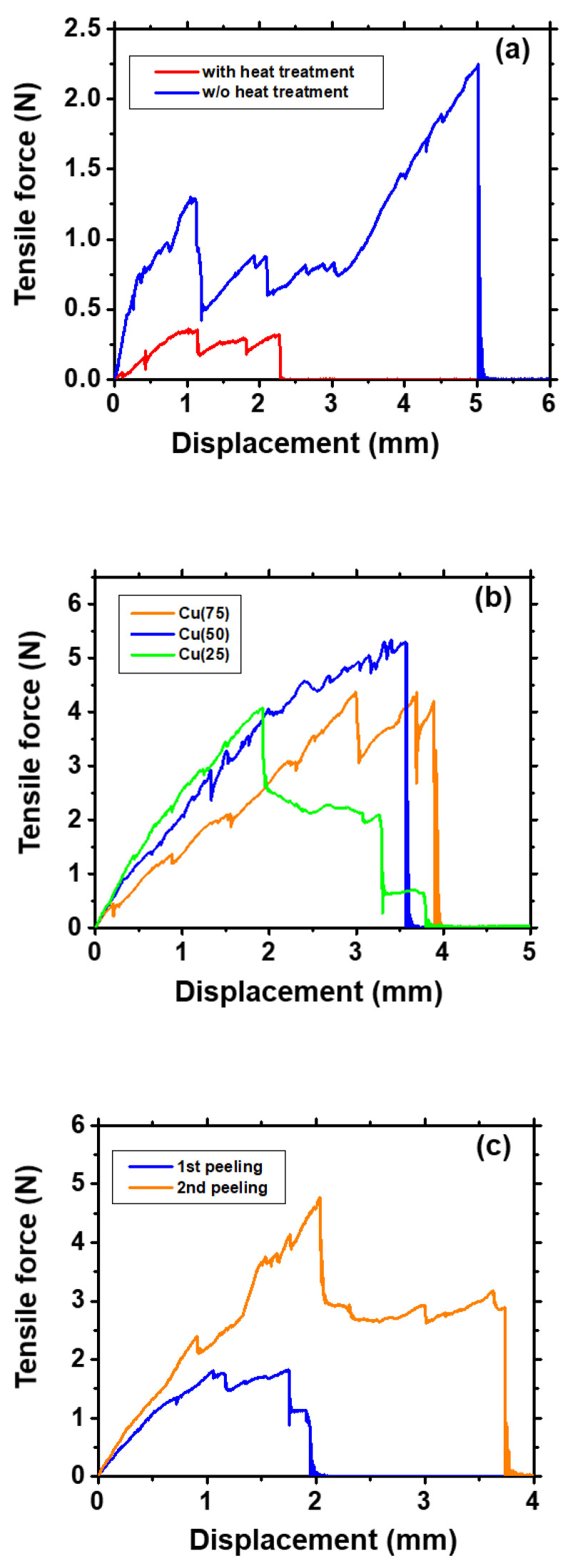
Results of the peeling force test for the samples in (**a**) group A, (**b**) group B, and (**c**) group C.

The results of the peeling force test, i.e., peeling energy, peeled area, and peeling energy per unit area (adhesion energy), are presented in Table 4. Figure 9a shows the relation between the displacement from the peeling start point and the tensile force for the samples in group A (α and β). The adhesion energy of α was approximately 1/10th of that of β. The existence of porosity owing to heat treatment resulted in the high peelability of α.

Figure 9b shows the relation between the displacement from the peeling start point and the tensile force for the samples in group B. The peeling energy of the samples in group B was higher than that of the samples in group A. A decrease in the Cu deposition time induced an increase in the adhesion of the DLC/Cu interface. A comparison of Cu (75), Cu (50), and Cu (25) revealed a decrease in the adhesion energy with an increase in the Cu deposition time.

Figure 9c shows the relation between the displacement from the peeling start point and the tensile force for the samples in group C. The peeling energy at the DLC I/Cu I interface was approximately equal to the peeling energy of Cu (25) in group B. Although the Cu I and II films were synthesized under identical conditions, the peeling energy at the Cu II/DLC II interface was calculated to be lower than that at the DLC I/Cu I interface. The results of the peeling force test in conjunction with the AFM observations revealed that a high surface roughness induced an increase in the peelability of the upper interfaces.

#### 3.3.2. Evaluation of Wear Resistance via BoD Test

**Figure 10:**
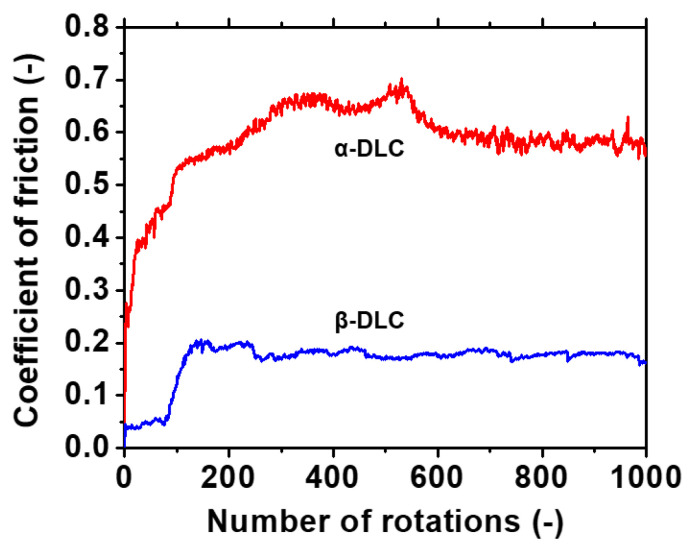
Results of the BoD test for the heat-treated (25 °C, 46% RH) and non-heat-treated (27 °C, 54% RH) samples in group A.

Figure 10 presents the results of the BoD test for the samples in group A. The friction coefficient of α was approximately 0.2 at the beginning of sliding, and it increased after 12 rotations. The friction coefficient after 200 rotations (0.6) was equivalent to the friction coefficient between the Si substrate and SUJ2. A substantial quantity of wear powder was generated in the initial stage of sliding. Therefore, the upper DLC II film was damaged after approximately 10 rotations; furthermore, the DLC I film underwent wear after 200 rotations because the Si substrate was exposed. The friction coefficient of β was maintained at approximately 0.05 in the initial stage of sliding. It increased after approximately 100 rotations owing to the wear of the upper DLC II film and Cu film; finally, the friction coefficient stabilized at 0.2 owing to the exposure of the DLC I film. The friction coefficient at the initial stage of sliding was as low as 0.1 because the Cu thin film was softer than the Si substrate. The DLC film underwent pressure relaxation owing to elastic deformation, thereby resulting in a lowering of the friction coefficient. Li et al. compared the results of the sliding tests for a DLC film formed on a Si substrate and a DLC film formed on a Cu substrate. They reported a decrease in the friction coefficient owing to the deformation of the soft Cu substrate [42]. These results indicated that heat treatment induced a marked decrease in the wear resistance.

**Figure 11:**
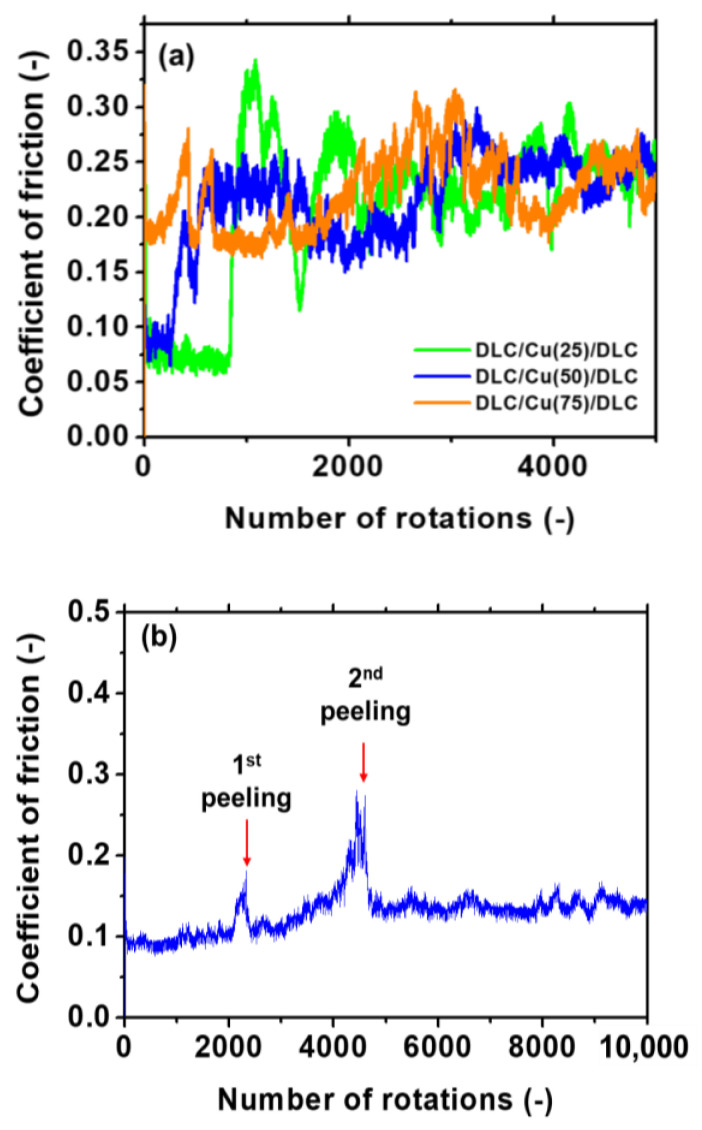
Results of the BoD test for the samples in (**a**) group B and (**b**) group C.

**Figure 12:**
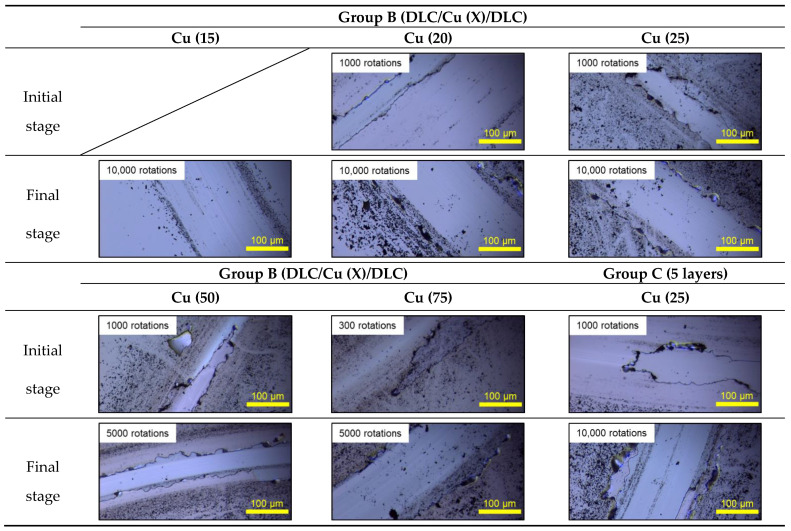
Images of the wear track of the films.

Figure 11 presents the results of the BoD test for the samples in groups B and C. The friction coefficient of DLC/Cu (75)/DLC was maintained at approximately 0.1 after approximately 300 rotations. Subsequently, it increased to approximately 0.2 owing to the generation of substantial wear debris and finally stabilized. Figure 12 shows the sliding surface after 300 and 5000 rotations. The presence of a storied structure at the boundary, where the upper DLC II/Cu I film did and did not undergo wear, resulted in exposure of the DLC I film.

DLC/Cu (50)/DLC and DLC/Cu (75)/DLC exhibited similar friction coefficients at the beginning of sliding. There was a gradual increase in the friction coefficient after approximately 1200 rotations to approximately 0.4. The exposed DLC I film was narrow and exhibited a wavy fracture surface. The gradual increase in the friction coefficient was attributed to the continuous generation of wear powder.

There was a significant increase in the friction coefficient of DLC/Cu (25)/DLC after approximately 1000 rotations. Subsequently, the friction coefficient exhibited an unstable state till approximately 6000 rotations. Finally, a stable friction coefficient of approximately 0.3 was obtained.

Figure 12 presents the sliding mark for the samples in group B, i.e., DLC/Cu (15), (20)/DLC, over 10,000 rotations. The DLC II film was abraded after the initial 1000 rotations. The DLC I film was exposed completely after 10,000 rotations because the upper DLC film was damaged. The quantity of black wear powder for these samples was lower than that for the other samples. The low quantity of the wear powder resulted in stable friction.

The initial friction coefficient for the samples in group C was approximately 0.1, which was similar to the friction coefficient of DLC/Cu (25)/DLC in group B. Two peaks were observed after 2200 and 4500 rotations in Figure 11b. The friction coefficient raised at 2200 rotations inferred that the DLC III film had worn out and Cu II layer was exposed. Then it went back to 0.1 immediately, which inferred the DLC II film was exposed due to the abrasion of Cu II layer. A similar phenomenon occurred at 4500 rotations. After that, the friction coefficient remained stable revealing that the entire 5-layer film was able to bear till 10,000 rotations. Figure 12 shows the sliding track at the end of the test after 10,000 rotations. A three-story structure was detected at the edge of the sliding mark. The DLC I film was eventually exposed owing to the gradual wear of DLC III/Cu II and DLC II/Cu I.

In samples DLC/Cu (20), (25)/DLC, the width of 1000 rotations were approximately 50 μm, and appeared approximately 165 μm after 10,000 rotations. The variation of time intervals between samples DLC/Cu (20)/DLC and DLC/Cu (25)/DLC were so small that no significant difference was observed. However, the width was approximately 50 μm in DLC/Cu (50)/DLC, approximately 170μm in DLC/Cu (75)/DLC after 5000 rotations. It is considered that the wear resistance decreased as the copper deposition time increased.

### 3.4. Fabrication of the 11-Layer DLC Film

**Figure 13:**
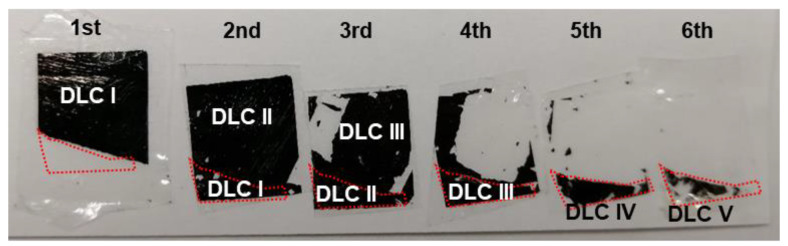
Appearance on the tapes of the 11-layer films after peeling.

Figure 13 shows the appearance of the films after peeling. Although the films were not uniformly peeled, the peelability of certain parts (within the range of the red dotted line) was five times of that of the upper film. The initially unpeeled area was peeled after the second peeling till the sixth peeling. The negligible differences in the peelability of each film set resulted in nonuniform peeling. These results indicated the dependence of the peelability on the Cu deposition time; in addition, they demonstrated the successful prevention of repeated seizures.

An increase in the number of films resulted in an increase in the production time. This was attributed to the requirement of cooling time. An additional increase in the number of peelings was expected to be difficult. It was inferred, considering the practical applications of the mold, that the shape of the molded product was dependent on the thickness of the deposited film on the mold surface; furthermore, the molding accuracy was dependent on the thickness of the DLC/Cu films.

## 4. Conclusions

The present study demonstrated the synthesis of multilayered DLC films via CVD and magnetron sputtering. The synthesized films with a DLC/Cu/DLC structure exhibited antiseizure properties that prevented the adhesion of instant adhesives. The appearance of the DLC film after peeling allowed the retention of wear resistance, and this indicated the successful development of a functional coating. The peeling interface of the film was subjected to SERS analysis. The results revealed that the deposition of the Cu film via magnetron sputtering induced the transformation of the DLC film at the polar interface into nanocrystal graphite.

Heat treatment induced an increase in the peelability. However, the existence of porosity at the Cu/DLC interface resulted in a significant decrease in the wear resistance. An increase in the Cu deposition time also induced an increase in the peelability. However, the load capacity in the shear direction of the Cu/DLC interface was lowered, and the occurrence of surface fracture owing to peeling was highly probable. This resulted in a decrease in the wear resistance. Both Cu films in the 5-layer sample (group C) were prepared simultaneously; however, the peeling energy at the upper Cu II/DLC II interface was lower than that at the DLC I/Cu I interface. All the DLC surfaces remained wear resistant for more than 1000 rotations in the BoD test.

The characteristics at the interface varied depending on the thickness of the Cu film and the stacking order of the DLC film. Therefore, it was possible to synthesize multilayered DLC films capable of withstanding more than 1000 cycles of sliding.

The effects of the distance between the substrate and target, bias voltage, and current during Cu sputtering on the peelability were not investigated in the present study. However, these parameters induced a variation in the energy of the sputtered particles, thereby affecting the graphitization of the DLC film at the polar interface. Therefore, the sputtering parameters can be considered as controlling factors for the peelability.

Future research on this subject may be conducted on the following topics:

(1) Thinning of DLC films.

(2) Determination of the correlation between the substrate temperature and the peelability.

(3) Control of the peelability using other parameters.

## Figures and Tables

**Table 1:** Conditions of cleaning, deposition, and sputtering.

Conditions	Ar Cleaning	DLC Deposition	Cu Sputtering
Source Gas	Ar	C_2_H_2_	Ar
Pressure (Pa)	2.0	3.0	2.0
Flow Rate (sccm)	20	20	20
Bias Frequency (kHz)	14.4	14.4	―
Bias Voltage (kVp)	−3.5	−4.0	−0.38
Current (A)	―	―	0.3
Time	30 min	20 min	15, 20, 25, 50, and 75 s

**Table 2:** Structures of various samples.

Group	No. Films	Structures *
A	3	DLC/Cu (300)/DLC
B	3	DLC/Cu (X)/DLC
C	5	DLC/Cu (25)/DLC/Cu (25)/DLC

* The numbers within parentheses after Cu indicate the deposition time (s) of the Cu film.

**Table 3:** Roughness and thickness of the films for the samples in group C.

Films	Roughness (nm)	Thickness (nm)
DLC III	5.14	180
Cu II	10.13	19
DLC II	5.36	178
Cu I	5.90	16
DLC I	1.94	124

**Table 4:** Peeling properties of the samples in each group.

Specimens			Peeling Energy (U)	Peeled Area (A)	Adhesion Energy (U/A)
Group A	α: With 500 °C heat treatment		5.21 × 10^−4^	8.90 × 10^−5^	5.86
	β: Without heat treatment		5.23 × 10^−3^	8.55 × 10^−5^	61.1
Group B	DLC/Cu (25)/DLC		11.5 × 10^−3^	6.02 × 10^−5^	191
	DLC/Cu (50)/DLC		9.66 × 10^−3^	7.00 × 10^−5^	138
	DLC/Cu (75)/DLC		2.83 × 10^−3^	5.44 × 10^−5^	52.0
Group C	DLC/Cu (25)/DLC/Cu (25)/DLC	First peeling	2.44 × 10^−3^	6.76 × 10^−5^	36.1
		Second peeling	9.70 × 10^−3^	6.15 × 10^−5^	158

## Data Availability

The data presented in this study are available on request from the corresponding author.
